# Pandora's box

**DOI:** 10.1192/bji.2018.26

**Published:** 2018-11

## Should we worry about medications in our water supplies?

We humans have been polluting our environment for centuries. The medical profession has been vociferous in expressing concerns about the effects of air and water pollution on our health and pressing for action to be taken to cleanse the environment. However, are we also culpable in contributing to environmental pollution?

Examination of wastewater treatment plants in the USA found drugs such as hydrochlorothiazide in all samples, and antihypertensives and carbamazepine in 90%, as well other drugs such as antibiotics and hormones as reported in other studies.

The World Health Organization (WHO) considered the matter and concluded that the very low concentrations of pharmaceuticals in drinking water were unlikely to present a risk to human health, although it recognised that the long-term effects are unknown. The WHO report suggested that future research looks into cost-effective methods to prioritise pharmaceuticals within the context of an overall risk assessment of the quality of drinking water and the effects on human health.

In the meantime, however, some countries have been concerned about improper disposal of drugs, such as down the sink or in the rubbish bin, as found in surveys in the USA, China and Serbia. The US Drug Enforcement Administration (DEA) ruled in 2014 to implement the Secure and Responsible Drug Disposal Act of 2010. In keeping with these regulations, pharmacies and healthcare facilities may register as designated collection sites for unwanted medications. By early 2016, there were 882 DEA-registered designated collectors across the USA. In the UK, the Pharmaceutical Services Negotiating Committee has worked with the Department of Health and Social Care, the Environment Agency and others to prepare guidance for pharmacies, which is set out in *Health Technical Memorandum 07-01: Safe Management of Healthcare Waste*.

Unless there is enough concern about our health and that of aquatic animals to make it a law that medicines are properly disposed of, it is unlikely that habits will change.

KinrysG., GoldA.K., WorthingtonJ.J.,  (2018) Medication disposal practices: increasing patient and clinician education on safe methods. Journal of International Medical Research, 46(3), 927–939.2932284510.1177/0300060517738681PMC5972255

World Health Organization (2011) Pharmaceuticals in Drinking-Water (WHO/HSE/WSH/11.05). WHO.

## Sewage analysis: a new toolkit in the research into illicit drug use

Sampling the sewage influent into a wastewater treatment plant has gained popularity in recent years as a scientific epidemiological method of monitoring geographical and temporal trends in illicit drug use. It allows the estimation of the quantity of drugs consumed by a community and their metabolites excreted in urine.

A Europe-wide network, Sewage Analysis CORe group Europe (SCORE), established in 2010, aimed to standardise the approaches used for wastewater analysis and coordinate international studies through the establishment of a common protocol of action. After a successful Europe-wide investigation in 2011, a large-scale investigation including 19 countries and 56 cities in Europe was launched in 2017.

The findings showed different patterns between European regions, as well as some temporal changes. Cocaine use was highest in Western and Southern European cities, and very low to negligible in most Eastern European cities. Amphetamine use was highest in Northern and Eastern Europe, and much lower in Southern European cities. However methamphetamine use, which had previously been low and concentrated in the Czech Republic and Slovakia, was now also found in Cyprus, Eastern Germany and Northern Europe.

The SCORE initiative and the findings of its research programme show that the wastewater analysis method provides a useful new toolkit for use in epidemiological illicit drug studies. It has the ability to monitor drug use, detecting new trends, which can inform the targeting of health programmes and policy initiatives to specific groups of people and the types of drugs they use.

European Monitoring Centre for Drugs and Drug Addiction (2018) Wastewater Analysis and Drugs: A European Multi-City Study. EMCDDA.

## Is our reasoning biased by our desires?

How objective are we when we consider our future prospects? Research shows that we are biased by optimism to disregard negative evidence. A human study using functional magnetic resonance imaging (fMRI) combined with computational and dynamic causal modelling to test for valence-guided belief formation (valence meaning *the subjective worth of* in keeping with Kurt Lewin's theory) found greater belief updates in response to good compared with bad news, and also identified the neurocircuitry mechanisms involved in this optimism bias.

The ventromedial prefrontal cortex (vmPFC) encoded the valence of updating in association with the valuation of rewards. It filtered the incoming signal in a valence-dependent manner and influenced the dorsomedial prefrontal cortex (dmPFC). Both the valence-encoding activity in the vmPFC and its influence on the dmPFC predicted the individual magnitude of the optimism bias.

The researchers argue that their findings demonstrate that belief formation, which is at the basis of human reasoning, is actually distorted to promote desired conclusions. Could it be that our brain protects us from spiralling into doom and gloom, given the often negative experiences in human existence?

KuzmanovicB., RigouxL. & TittgemeyerM. (2018) Influence of vmPFC on dmPFC predicts valence-guided belief formation. Journal of Neuroscience, DOI:10.1523/JNEUROSCI.0266-18.2018.PMC659615130104337

## Whether you get on with things or put them off for later is down to your amygdala

We humans vary in our ability to initiate self and emotional control, differences that have been described by ‘action control theory’. Researchers claim that this variability has a neural basis. They carried out MRI scans in 264 women and men, assessing the volume of individual brain regions and the functional connectivity between them. Subjects were asked to complete a survey measuring their ability to execute action control. The differences in action control were related to variations in brain structure and resting state connectivity. The amygdala and the dorsal anterior cingulate cortex (ACC) have been identified previously as the key areas involved in action control. The amygdala assesses situations and their outcomes, warning about potential negative consequences. The role of the dorsal ACC is to use this information to select appropriate actions, and in the process it also suppresses competing actions and emotions in order to ensure successful completion of the selected action.

The researchers found that decision-related action orientation (AOD) scores were associated with the size of the amygdala. The lower the AOD score, the larger the amygdala volume and the weaker the resting connectivity between the amygdala and the dorsal ACC.

SchlüterC., FraenzC., PinnowM.,  (2018) The structural and functional signature of action control. Psychological Science, DOI:10.1177/0956797618779380.30118388

## The ‘rosehip neuron’ that sets us apart from other non-primate animals

Our superior brain ability gives humans an advantage over other animals. This concerns more specifically the top layer of our brains, which is much larger than those of other animals relative to our bodies, and much more complex than that of any other creature. This part of our brain is thought to be responsible for important functions such as consciousness, which are considered to be unique to the human species. However, the specific substrate of this higher brain ability has remained elusive so far.

Research groups from Hungary and the USA combined their varied expertise in neuroscience and claim to have unravelled the mystery, at least in part, identifying a novel neuronal structure in humans, which is missing in other non-primate animals. They examined sections from the top layer of the cortex of the brains of two men who had died in their 50s and had donated their bodies to research. Using evidence from transcriptomics, anatomy and neuronal physiology, they identified a specialised GABAergic subtype in the human cortex. This consists of a group of human interneurons with characteristic anatomical features (large rosehip-like axonal boutons and compact arborisation), as well as an immunohistochemical profile with a specific molecular marker signature, none of which have ever been described in rodents. These rosehip neurons are positioned in a way that enables powerful local neural control.

BoldogE., BakkenT.E., HodgeR.D.,  (2018) Transcriptomic and morphophysiological evidence for a specialized human cortical GABAergic cell type. Nature Neuroscience, DOI:10.1038/s41593-018-0205-2.PMC613084930150662

## Being daring or cowardly is down to our hippocampus

The hippocampus is a key area in the brain with very important functions in memory/cognition (dorsal area) and emotion (ventral area), exerted through specific rhythmic neural oscillating pacemakers. Type 1 theta oscillations occur in the dorsal hippocampus in animals during movement and exploration, while type 2 theta oscillations in the ventral hippocampus appear in emotionally charged situations such as the presence of a predator.

New animal research, a collaboration between neuroscience centres in Sweden and Brazil, shows that the hippocampus may also be the determinant of how daring we can be in the face of danger. According to the scientists, this is down to a group of interneurons, called oriens-lacunosum moleculare (OLM) cells, located in the ventral hippocampus. The researchers showed that optogenetic activation of OLM interneurons drives the generation of type 2 theta oscillations, which are associated with increased risk-taking behaviour in response to predator odour (in animals).

It has previously been shown that OLM cells are the gatekeepers to memories in the hippocampus and that they are sensitive to pharmacological treatment. If these findings also apply to humans, could this pave the way for the development of possible pharmacological treatments for phobic anxiety disorders?

MikulovicS., RestrepoC.E., SiwaniS.,  (2018) Ventral hippocampal OLM cells control type 2 theta oscillations and response to predator odour. Nature Communications, DOI: 10.1038/s41467-018-05907-w.PMC612890430194386

